# Negative tension controls stability and structure of intermediate filament networks

**DOI:** 10.1038/s41598-021-02536-0

**Published:** 2022-01-07

**Authors:** Ehud Haimov, Michael Urbakh, Michael M. Kozlov

**Affiliations:** 1 School of Physics and Astronomy, Raymond and Beverley Sackler Faculty of Exact Sciences, Tel-Aviv University, 69978 Tel-Aviv, Israel; 2 School of Chemistry, Raymond and Beverley Sackler Faculty of Exact Sciences, Tel-Aviv University, 69978 Tel-Aviv, Israel; 3 Department of Physiology and Pharmacology, Sackler Faculty of Medicine, Tel-Aviv University, 69978 Tel-Aviv, Israel

**Keywords:** Biophysics, Physics

## Abstract

Networks, whose junctions are free to move along the edges, such as two-dimensional soap froths and membrane tubular networks of endoplasmic reticulum are intrinsically unstable. This instability is a result of a positive tension applied to the network elements. A paradigm of networks exhibiting stable polygonal configurations in spite of the junction mobility, are networks formed by bundles of Keratin Intermediate Filaments (KIFs) in live cells. A unique feature of KIF networks is a, hypothetically, negative tension generated in the network bundles due to an exchange of material between the network and an effective reservoir of unbundled filaments. Here we analyze the structure and stability of two-dimensional networks with mobile three-way junctions subject to negative tension. First, we analytically examine a simplified case of hexagonal networks with symmetric junctions and demonstrate that, indeed, a negative tension is mandatory for the network stability. Another factor contributing to the network stability is the junction elastic resistance to deviations from the symmetric state. We derive an equation for the optimal density of such networks resulting from an interplay between the tension and the junction energy. We describe a configurational degeneration of the optimal energy state of the network. Further, we analyze by numerical simulations the energy of randomly generated networks with, generally, asymmetric junctions, and demonstrate that the global minimum of the network energy corresponds to the irregular configurations.

## Introduction

Formation of dynamic networks is ubiquitous in soft matter systems and cytoplasm of biological cells^1–4^. Of a special interest are networks, whose vertices, referred below to as the junctions, are able to move along the network edges. Two examples of the previously investigated networks with mobile junctions are the two-dimensional soap froths^3^ called below the soap film networks, and the polygonal networks of membrane tubules, which form a significant part of one of the most crucial intracellular organelles, the endoplasmic reticulum (ER)^4^.

**Figure 1:**
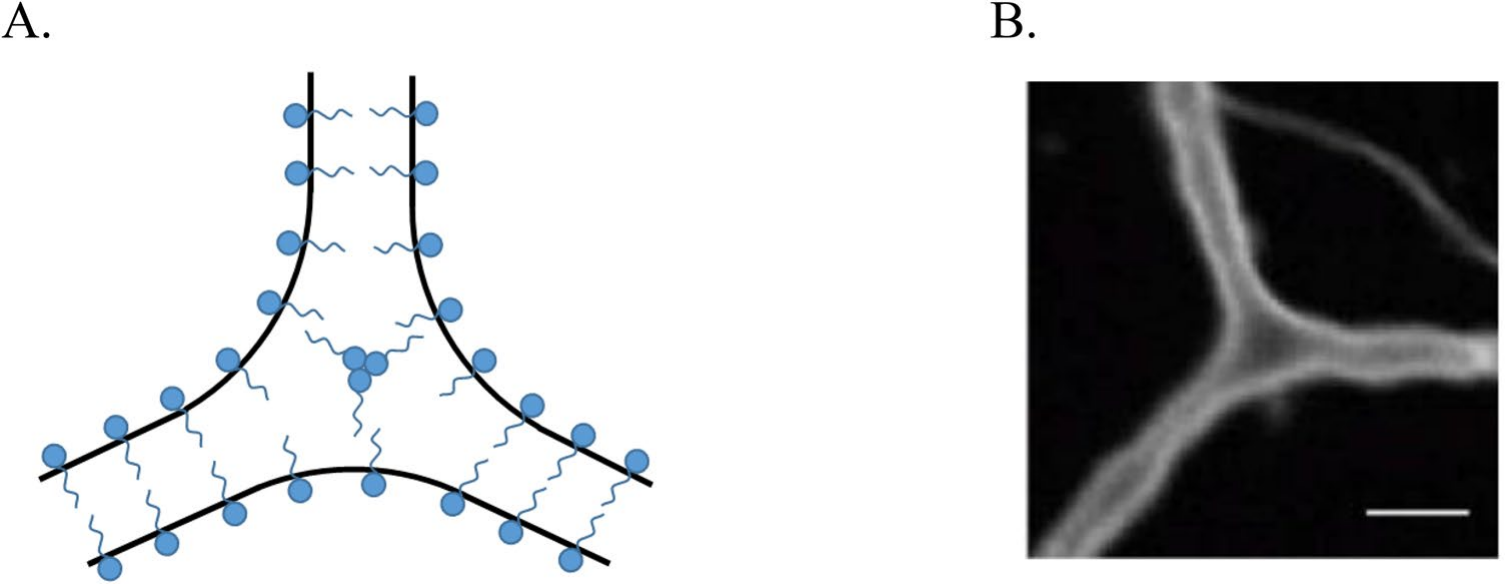
Movable network junctions. (**A**) A sketch illustrating a three-way junction in a planar soap froth network. (**B**) Three-way junction between dilated ER tubules formed with Xenopus egg membranes^6^ (Scale bar: 3 μm).

The junction mobility is enabled by the ability of the network edges to freely flow through the junctions, which, in turn, requires a direct merger of the edge ends within the junctions and an effective lateral fluidity of the edge material. This condition is, obviously, satisfied for soap film networks, whose edges and junctions are filled by aqueous solutions and covered by fluid soap monolayers (Fig. 1A). In the ER networks, lipid bilayers serving as a base of the tubular membranes behave as a two-dimensional fluid and are smoothly inter-merged within the junctions (Fig. 1B)^5^.

The prominent feature of the soap film and ER tubular networks is their dynamic behavior and instability. The soap film networks exhibit collapse or unlimited expansion of their polygonal elements, which is mediated by the movement and fusion of the network junctions^3^. Similarly, the ER tubular networks undergo a perpetual remodeling through collapse of their polygonal unit-cells, the phenomenon called the ring closure^4^.

The factor driving the instability of the ER- and soap- film networks is the positive tension imposed on the network elements. In the ER, stretching forces applied by the intra-cellular force-generating machinery create tension in the network’s tubular membranes^7,8^. In soap film networks, the tension originates from the surface tension of the soap solution-air interface. The positive tension tends to minimize the overall length of the network edges, which drives the network remodeling mediated by movement of the junctions^3,8^. Substantiating the experimental observations^5,8^, computational simulations of networks with mobile junctions and positive tension recovered the temporal evolution of the system consisting in expansion and collapse of the network unit-cells^9^. The dynamic structural rearrangements of the ER and soap film networks drive the eventual degradation of the network unless a counter-process, such as a de novo tubule generation and fusion in ER^5^, restores the network junctions and constituent elements.

A question arises whether a negative tension favoring an increase rather than a decrease of the overall length of the network edges would prevent the degradation and support stabilization of a network with mobile junctions. While, to the best of our knowledge, mobile junction networks subject to negative tensions have not been previously investigated, at least one paradigm of this kind of network exists within live cells and provides a motivation for such analysis. Those are the networks of bundles formed by intermediate filaments (IFs), which represent a part of a sophisticated system of intracellular polymers called the cytoskeleton^10,11^.

The protein composition of an IF depends of the cell type and the intracellular localization^12^. Independently of the specific protein composition, different IFs have common structural features^13^. They are built through polymerization of 65 nm long subunits composed of eight apolar tetramers, each formed by two dimers joined together in an anti-parallel fashion^13^. IFs possess a substantial rigidity with respect to bending^14^. Therefore, they exhibit properties of semi-flexible 10 nm thick polymers, whose persistence lengths substantially exceed the size of a single subunit and vary in a broad range between hundreds of nanometers to a few microns^14^. While the physical principles of formation and functioning of IFs have been thoroughly addressed^14^, several essential aspects of IF intracellular organization have remained less-understood. One proposed function of IF networks is a contribution to cell mechanical stability and integrity^15^.

We will base our modeling on specific data obtained for networks of IFs consisting of a particular protein, Keratin, and referred to as the Keratin Intermediate Filaments (KIFs)^16^. Like other intermediate filaments, KIFs exhibit properties of 10 nm thick semi-flexible polymers, which self-organize into bundles^16–18^ with a cross-sectional diameter of about 100 nm^19,20^. These bundles self-assemble into networks^16^ whose edges are formed by the bundles themselves whereas the vertices are represented by the three-way junctions between the bundle ends.

An intra-cellular KIF network can be seen as consisting of two parts: a peripheral network, which fills the space between the cell periphery and the nucleus, and a central network covering the cell nucleus (for a fluorescence microscopy time-lapse image of KIF network in live cells, see ref.^16^).

The peripheral network is dynamic. Keratin filaments are nucleated at the cell edge and undergo a persistent flow towards the interface between the cytoplasm and the nucleus, where matured bundles concentrate and, apparently, undergo disassembly^16^.

The central network, in contrast to the peripheral one, is stationary. It does not exhibit any dynamic behavior except for limited fluctuations in the positions of the network junctions and bundles, as seen in fluorescence microscopy time-lapse imaging of KIF network in live cells (see ref.^16^). Structurally, the central network consists of approximately polygonal unit-cells of different types with a typical size of several microns. The central network is bounded by the nucleus-cytoplasm interface region.

**Figure 2:**
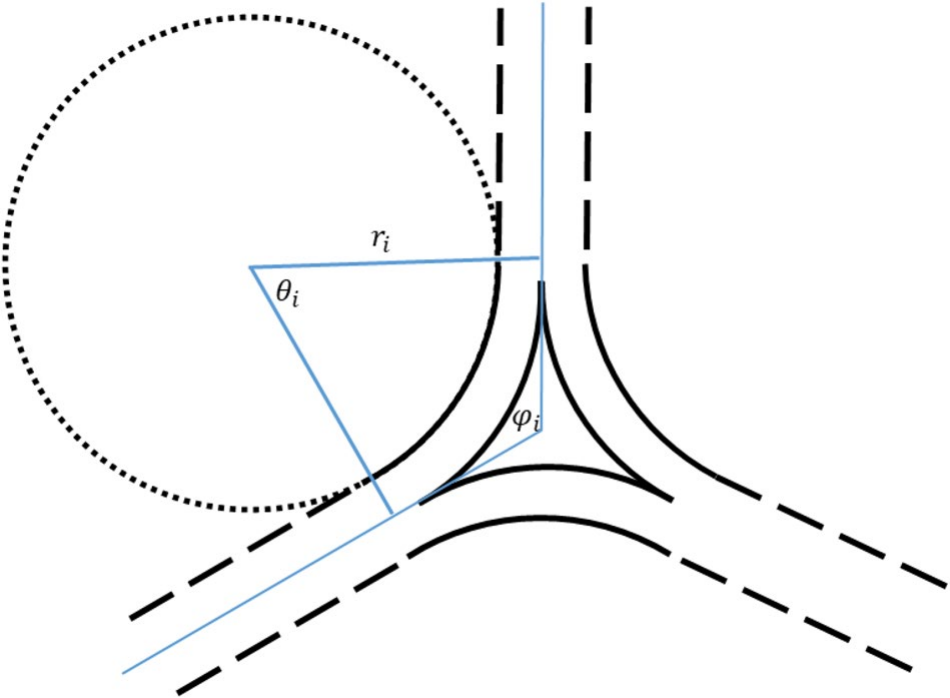
A sketch illustrating the structure of a three-way junction and the notations used in the model.

KIF network junctions are mobile, as demonstrated by observations of the junction behavior in the dynamic peripheral network^16^. A direct merger of the bundle ends within the junctions, as required for the junction mobility, is supported by the fact that no molecular linkers are known to be necessary for formation and functioning of KIF networks. The structure of a junction resulting from a pair-wise merger of three bundle ends is illustrated in (Fig. 2) and involves the splitting of each bundle into two branches.

The second condition required for the junction mobility is an effective lateral fluidity of the bundles which is, in essence, the ability of the filaments constituting the bundles to freely slide with respect to each other. Given that the filaments are not interlinked by either direct or indirect chemical bonds^19^, there should be no obstacles for such sliding.

The tension in the central KIF network is expected to be negative rather than positive for the following reason. It is sensible to assume that the interface zone between the peripheral and central networks serves as an effective reservoir of material for the bundles in the central network, as seen in fluorescence microscopy time-lapse imaging of KIF network in live cells (see ref.^16^). Indeed, according to the observations^16^, this zone forms as a result of concentration and disintegration of the KIF bundles arriving from the cell periphery and must, therefore, represent a pool of filament elements which should be freely exchangable with the central network. Since the formation of longer filaments and their subsequent lateral association into bundles are energetically favorable, the chemical potential of the bundles within the KIF central network must be lower than that in the effective reservoir, which, in turn, results in a negative network tension.

Summarizing, we propose that the central network of KIF is a paradigm of a stable two-dimensional network, whose edges made of KIF bundles are connected by mobile three-way junctions representing the network vertices and exposed to a negative tension.

Another property of the central KIF network, which can, potentially, contribute to the network stability, is the resistance of the network junctions to deviations from the symmetric configuration, in which all three angles between each pair of adjacent bundles merged within the junction are equal $\frac{2\pi }{3}$. These deviations, referred below to as the junction folding, generally accompany, in addition to the junction movement, the network dynamic rearrangements. The junction resistance to the folding must originate from the bending rigidity of the bundle branches constituting the junction (Fig. 2). Since the bending moduli of KIFs and their bundles are substantial^14^, the KIF junction resistance to the folding must be significant and may, therefore, impede the network dynamics.

Here we analyze the structure and stability of two-dimensional networks subject to negative tensions and inter-linked by mobile three-way junctions of substantial but finite resistance to folding. Our analysis includes two steps. First, we analytically examine a simplified case of a regular hexagonal network. We demonstrate that a negative tension is indeed mandatory for the existence of a highly degenerated family of stable equilibrium network configurations. For these configurations, we determine an optimal average density of the network junctions and compare it on a semi-quantitative level with the experimental observations of KIF networks. Second, we demonstrate by numerical simulations the existence of irregular network configurations, whose energies are significantly lower than the lowest energies of a regular hexagonal network. These irregular configurations are characterized by, generally, asymmetric junctions. Importantly, the average junction density of an optimal irregular network configuration is close to that found for the optimal configurations of a regular hexagonal network.

## Results

We consider the central KIF network as a polygonal mesh of bundles inter-connected by three-way junctions. The network is free to exchange KIF material with a surrounding reservoir. The total network area, $A$, is sufficiently large so that the radius of the network boundary with the reservoir, $R$, significantly exceeds the typical dimension, $b$, of the network unit-cell, $R \gg b$.

Our goal is to analyze the conditions of existence and stability of the network equilibrium states by minimizing the network free energy, $F$, determined as the thermodynamic work needed to form the network out of the reservoir material. We consider the energy, $F$, to consist of two contributions, the energies of bundles, $F_{B}$, and of junctions, $F_{j}$, such that $F = F_{B} + F_{j}$.

We start by introducing, separately, each energy contribution and the related system parameters. Next, we analyze the stability and density of networks with symmetric junctions and finish by showing a numerical analysis of networks with asymmetric junctions.

### Bundle energy

Formation of the network bundles from the reservoir material is accompanied by an energy release related to KIF polymerization and bundling so that the network bundle energy, $$F_{B}$$, is negative. The energy, $$F_{B}$$, related to the bundle unit length is equal to the network tension denoted by, − $$\gamma$$, such that $$\gamma$$ is positive and represents the absolute value of the tension. Hence, the bundle energy, $$F_{B}$$, can be expressed through the total length of the network bundles, $$L, $$ by

1 $$ F_{B} = - \gamma L. $$

### Junction energy

The total junction energy, $F_{j}$, is assumed to be the sum of energies of individual junction energies, $f_{j}$, implying that there is no interaction between the junctions. Therefore, we consider here a single junction, whose overall configuration is determined by the three angles, $\phi_{i}$, between the main axes of the adjacent bundles in the junction (Fig. 2). A junction will be referred to as symmetric if all the angles $\phi_{i}$ are equal to each other (and to $\frac{2\pi }{3}$).

We consider the bundle branches forming a junction to have shapes of circular arcs of, in general, different radii of curvature, $r_{i}$, different arc angles, $\theta_{i}$, and, consequently, different arc lengths, $l_{{{\text{S}}i}}$ (the subscript $i$ denoting the number of the arc). The arc angles, $\theta_{i}$, are related to the inter-bundle angles, $\phi_{i}$ (Fig. 2), which are expressed through the following geometrical relationships,

2 $$ \begin{aligned} r_{1} \tan \left( {\frac{{\theta_{1} }}{2}} \right) & = r_{2} \tan \left( {\frac{{\theta_{2} }}{2}} \right) = r_{3} \tan \left( {\frac{{\theta_{3} }}{2}} \right), \\ \theta_{i} & = \pi - \phi_{i}. \\ \end{aligned} $$

The single-junction energy, $f_{{\text{j}}}$, will be related to the reference state preceding the junction formation, in which the bundle ends are not split into branches. We consider two contributions to $f_{{\text{j}}}$, the energy of the bundle splitting, $f_{{\text{S}}}$, and the bending energy of the branches resulting from the arc formation, $f_{{\text{B}}}$, so that, $f_{{\text{j}}} = f_{{\text{S}}} + f_{{\text{B}}}$. We calculate the dependence of the two energy contributions, $f_{{\text{S}}}$ and $f_{{\text{B}}}$, on the arcs′ radii, $r_{i}$, and lengths, $l_{{{\text{S}}i}}$, and determine the optimal structure and energy of the junction by minimizing $f_{{\text{j}}}$ with respect to these values.

We assume the energy of the bundle end splitting to be proportional to the length of the split region so that the total energy of splitting can be expressed through the three arc lengths by

3 $$ f_{{\text{S}}} = \frac{1}{2} \varepsilon_{{\text{s}}} \left( {l_{{{\text{S1}}}} + l_{{{\text{S2}}}} + l_{{{\text{S3}}}} } \right), $$

where $\varepsilon_{S}$ is the splitting energy per unit length.

Considering the bending energy per arc unit length to be quadratic in the arc curvature, $\frac{1}{r}$, which implies that the bundle branches have no intrinsic tendency to bend, the total bending energy of one arc, $f_{Bi}$ can be presented as

4 $$ f_{Bi} = \frac{1}{2} \kappa_{{\text{s}}} \frac{1}{{r_{i}^{2} }} l_{{{\text{Si}}}} , $$

where ${ }\kappa_{{\text{s}}}$ is the arc bending modulus. Based on (Eqs. 3, 4) and the relationship, $l_{{{\text{S}}i}} = r_{i} \theta_{i}$, the junction energy can be written as

5 $$ f_{{\text{j}}} = \frac{1}{2}\left( {\frac{{\kappa_{{\text{s}}} \theta_{1} }}{{r_{1} }} + r_{1} \theta_{1} \varepsilon_{{\text{s}}} } \right) + \frac{1}{2}\left( {\frac{{\kappa_{{\text{s}}} \theta_{2} }}{{r_{2} }} + r_{2} \theta_{2} \varepsilon_{{\text{s}}} } \right) + \frac{1}{2}\left( {\frac{{\kappa_{{\text{s}}} \theta_{3} }}{{r_{3} }} + r_{3} \theta_{3} \varepsilon_{{\text{s}}} } \right). $$

Using the geometrical relationships (Eq. 2) and accounting for the condition $\mathop \sum \limits_{i = 1}^{3} \phi_{i} = 2\pi$, the energy in (Eq. 5) can be presented as a function of two inter-bundle angles, e.g. $\phi_{1}$ and $\phi_{2}$, and the radius of one of the arcs, e.g. $r_{1}$. Minimizing the resulting expression for $f_{{\text{j}}}$ with respect to $r_{1}$, we obtain the dependences for the optimal arc radius, $r_{1}^{*}$, and energy, $f_{{\text{j}}}^{*}$, on the inter-bundle angles, $\phi_{1}$ and $\phi_{2}$.

6 $$ r_{1}^{*} = \lambda \sqrt {\frac{{\left( {\pi - \phi_{1} } \right) + \left( {\pi - \phi_{2} } \right)\frac{{\tan \left( {\frac{{\phi_{1} }}{2}} \right)}}{{\tan \left( {\frac{{\phi_{2} }}{2}} \right)}} + \left( {\pi - \phi_{1} - \phi_{2} } \right)\frac{{\tan \left( {\frac{{\phi_{1} }}{2}} \right)}}{{\tan \left( {\frac{{\phi_{1} + \phi_{2} }}{2}} \right)}}}}{{\left( {\pi - \phi_{1} } \right) + \left( {\pi - \phi_{2} } \right)\frac{{\tan \left( {\frac{{\phi_{2} }}{2}} \right)}}{{\tan \left( {\frac{{\phi_{1} }}{2}} \right)}} + \left( {\pi - \phi_{1} - \phi_{2} } \right)\frac{{\tan \left( {\frac{{\phi_{1} + \phi_{2} }}{2}} \right)}}{{\tan \left( {\frac{{\phi_{1} }}{2}} \right)}}}}} $$

7 $$ f_{{\text{j}}}^{*} \left( {\phi_{1} ,\phi_{2} } \right) = \frac{1}{2\pi }f_{{\text{j}}}^{0} \left[ {\left( {\pi - \phi_{1} } \right)\left( {\frac{1}{{\rho_{1}^{*} }} + \rho_{1}^{*} } \right) + \left( {\pi - \phi_{2} } \right)\left( {\frac{1}{{\rho_{1}^{*} }}\frac{{\tan \left( {\frac{{\phi_{1} }}{2}} \right)}}{{\tan \left( {\frac{{\phi_{2} }}{2}} \right)}} + \rho_{1}^{*} \frac{{\tan \left( {\frac{{\phi_{2} }}{2}} \right)}}{{\tan \left( {\frac{{\phi_{1} }}{2}} \right)}}} \right) + \left( {\pi - \phi_{1} - \phi_{2} } \right)\left( {\frac{1}{{\rho_{1}^{*} }}\frac{{\tan \left( {\frac{{\phi_{1} }}{2}} \right)}}{{\tan \left( {\frac{{\phi_{1} + \phi_{2} }}{2}} \right)}} + \rho_{1}^{*} \frac{{\tan \left( {\frac{{\phi_{1} + \phi_{2} }}{2}} \right)}}{{\tan \left( {\frac{{\phi_{1} }}{2}} \right)}}} \right)} \right] , $$

where

8 $$ f_{{\text{j}}}^{0} = \pi \sqrt {\kappa_{{\text{s}}} \varepsilon_{{\text{s}}} } , $$

is the intrinsic energy scale of the junction, and $\rho_{1}^{*} = \frac{{r_{1}^{*} }}{\lambda }$ is the normalized optimal arc radius with

9 $$ \lambda = \sqrt {\frac{{\kappa_{{\text{s}}} }}{{\varepsilon_{{\text{s}}} }}} , $$

being the intrinsic length scale. Numerical analysis of the optimal junction energy, presented by (Eq. 7), shows that it is minimal for a symmetric junction with $\phi_{1} = \phi_{2} = \frac{2\pi }{3}.$

For small deviations from the symmetric junction configuration referred to as the junction folding, the junction energy can be approximated by an expression accounting for contributions of the first non-vanishing order in the angle deviations from $\frac{2\pi }{3},$

10 $$ f_{{\text{j}}}^{*} \left( {\phi_{1} ,\phi_{2} } \right) \approx f_{{\text{j}}}^{0} \left( {1 + \frac{4}{9}\left( {\left( {\phi_{1} - \frac{2\pi }{3}} \right)^{2} + \left( {\phi_{2} - \frac{2\pi }{3}} \right)^{2} + \left( {\phi_{1} - \frac{2\pi }{3}} \right)\left( {\phi_{2} - \frac{2\pi }{3}} \right)} \right)} \right). $$

According to (Eq. 10), the energy of a symmetric junction is equal to $f_{{\text{j}}}^{0}$ (Eq. 8). The three arc radii of a symmetric junction, as follows from (Eq. 6), are equal to the characteristic length, $r^{*} = \lambda$ (Eq. 9). An effective junction rigidity of the folding deformations is proportional to $f_{{\text{j}}}^{0}$.

### Hexagonal network with symmetric junctions

Here we consider a network consisting of bundles connected by symmetric junctions. The energy of such a network can be presented, according to (Eqs. 1, 10), as

11 $$ F_{{\text{N}}} = N_{{\text{j}}} f_{{\text{j}}}^{0} - \gamma L, $$

where $f_{{\text{j}}}^{0}$ is given by (Eq. 8).

**Figure 3:**
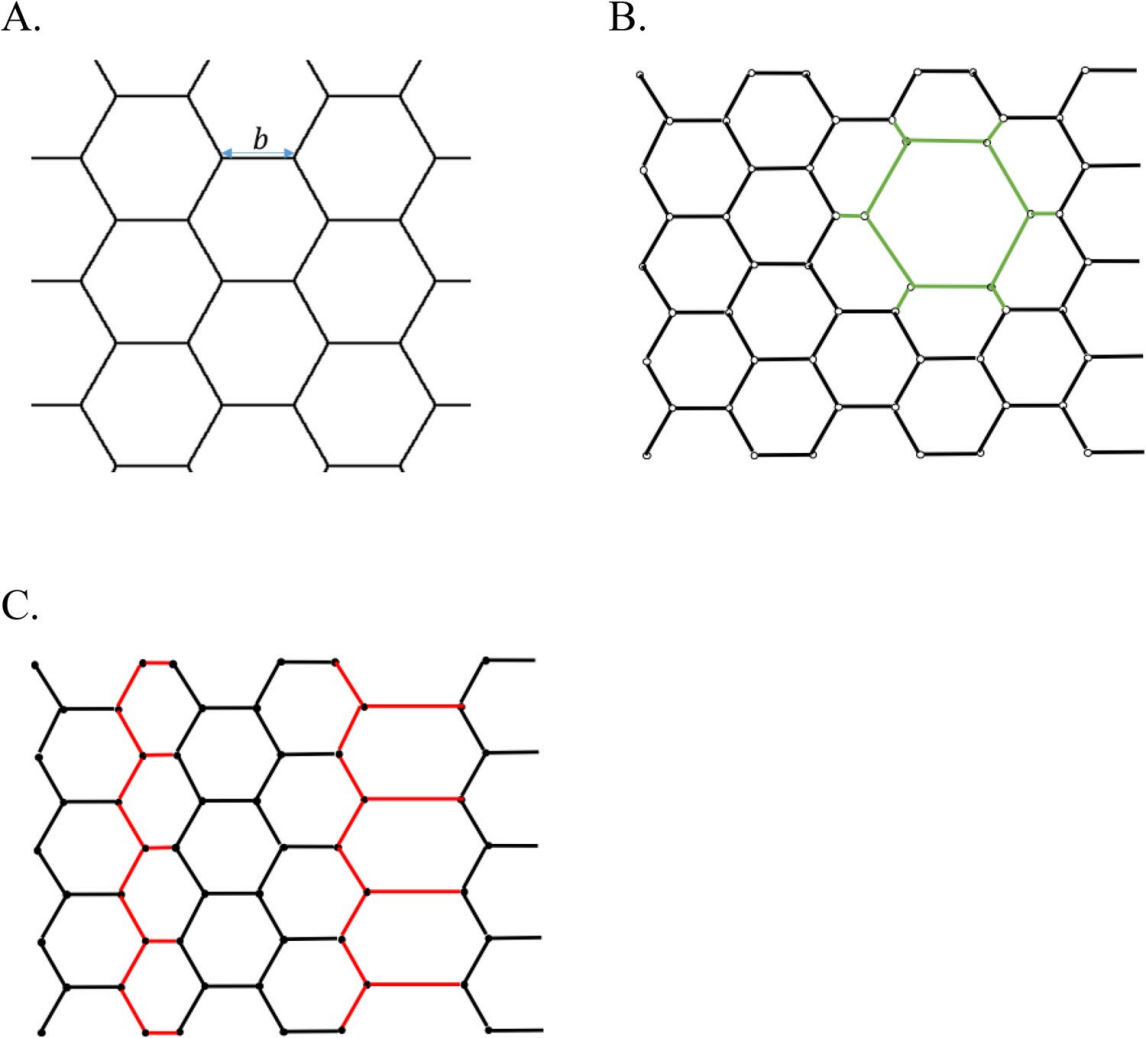
(**A**) A homogenous hexagonal network of hexagon side length denoted by $b$. (**B**) An example of an energy-preserving isotropic transformation induced by expansion or contraction of an isotropic structural element denoted by the green-colored edges. (**C**) An example for an energy-preserving telescopic transformation induced by expansion/contraction of a linear structural elements denoted by the red-colored edges.

Our goal is to determine the network equilibrium configuration, and analyze its stability and optimal density. We start with the analysis of a homogeneous network whose unit-cells have shapes of identical hexagons with the length $b$ of a hexagon side (Fig. 3A).

#### Network equilibrium and stability

The network is in equilibrium if the total force and torque acting on each junction and bundle of the network vanish. The only forces acting in the system originate from tension in the bundles and act along the bundles. The total force applied to each junction vanishes due to the symmetry (Fig. 3A). The symmetry of the junctions is also the reason for vanishing torques. Altogether, the homogeneous hexagonal network with symmetric junctions is in a mechanical equilibrium.

To analyze the stability of this equilibrium state, we calculated the change in the network energy upon an infinitesimal displacement of a single junction in a random direction (see Supplementary Note 1). We found that the network is stable against such displacements under the following condition:

12 $$ f_{{\text{j}}}^{0} > \frac{9}{20} \gamma b . $$

This equation reflects the interplay between the energy changes due to the negative tension, -$\gamma$, and the junction rigidity to folding, $f_{{\text{j}}}^{0}$. The negative tension favors the junction displacement due to the related increase of the overall bundle length, $L$. The elastic energy of the junction (Eq. 10) resists the displacements because of the related deviation of the junction from the symmetric conformation.

#### Optimal network density

The ratio of the total number of the network junctions,$N_{{\text{j}}}$, to the total network area, $A$, will be referred to as the network density, $\sigma = \frac{{N_{{\text{j}}} }}{A}$. To find the optimal value of $\sigma$ we have to minimize the total network energy (Eq. 11) with respect to the junction number $N_{{\text{j}}}$.

Using the smallness of the network unit-cells compared to the overall network $b \ll R$, we can neglect the contributions to the total bundle length from the hexagons, which are directly adjacent to the network boundary. In this approximation, the total bundle length, $L,$ can be expressed as $L = \frac{3}{2}bN_{{\text{j}}}$, while the network area can be presented as $A = \frac{{3\sqrt {3 } }}{4}b^{2} N_{{\text{j}}}$. Using these two relationships, we obtain $L = \sqrt {\sqrt 3 A N_{{\text{j}}} }$ and the energy $F_{{\text{N}}}$ (Eq. 11) can be rewritten as

13 $$ F_{{\text{N}}} = N_{{\text{j}}} f_{{\text{j}}}^{0} - \gamma \sqrt {\sqrt 3 A N_{{\text{j}}} } . $$

Minimization of (Eq. 13) results in the optimal network density,

14 $$ \sigma^{*} = \frac{{N_{{\text{j}}}^{*} }}{A} = \frac{\sqrt 3 }{4} \left( {\frac{\gamma }{{f_{{\text{j}}}^{0} }}} \right)^{2} . $$

The side length of the network unit-cell, $l_{{\text{c}}}$, corresponding the optimal density (Eq. 14) is given by

15 $$ l_{{\text{c}}} = \frac{4}{3}\frac{{f_{{\text{j}}}^{0} }}{\gamma }. $$

This side length serves as a characteristic length for the network structure, whereas the total network bundle length, $L^{*}$, is given by

16 $$ L^{*} = \frac{2}{\sqrt 3 }\frac{1}{{l_{{\text{c}}} }}A. $$

The corresponding minimal value of the network energy is given by

17 $$ F_{{\text{N}}}^{*} = - \frac{1}{\sqrt 3 }\frac{\gamma }{{l_{{\text{c}}} }}A . $$

The network tension can be evaluated based on the bundling energy of KIF filaments^21^ as $\gamma \approx - 170 \times 10^{3} {\text{k}}_{{\text{B}}} {\text{T }}$ µm^−1^. Based on the images of KIF central networks, the unit-cell length is about $l_{{\text{c}}} \approx 1$ µm. Using these parameter values, the free energy per unit area of the network optimal configuration is, approximately $\frac{{F_{{\text{N}}}^{*} }}{A} \approx - 9.81 \times 10^{4} {\text{k}}_{{\text{B}}} {\text{T}}$ µm^−2^.

#### Degeneracy of the optimal network state

The homogeneous hexagonal network with symmetric junctions described above is not the only configuration characterized by the minimal energy, $F_{{\text{N}}}^{*}$ (Eq. 17). As we show below, there are continuous transformations of the initial homogeneous configuration, that keep constant the overall network bundle length, $L^{*}$, the junction number, $N_{{\text{j}}}^{*}$, and do not disturb the symmetric state of the junctions. Hence the network configurations obtained through these transformations have the same optimal energy, $F_{{\text{N}}}^{*}$ (Eq. 17) as the homogeneous hexagonal configuration. This means that the network’s lowest energy state is energetically degenerated. Below we describe these transformations and analyze the dependence of the number of the equal energy states on the junction number, $N_{{\text{j}}}^{*}$.

We found two kinds of such network transformations which will be referred to as the isotropic and the telescopic transformations (see Fig. 3B, C).

To describe the isotropic transformation, we define a network structural element consisting of one hexagonal unit-cell with six network edges emerging from the hexagon vertices in the radial directions (Fig. 3B) and refer to it as the isotropic structural element. For each isotropic structural element, in the initial state the lengths of the hexagon sides and those of the radial edges are equal to each other and to $l_{{\text{c}}}$. The essence of the transformation is an isotropic expansion or contraction of the structural element. The expansion (contraction) leads to increase (decrease) of the hexagon side length from $l_{{\text{c}}}$ to $2l_{{\text{c}}}$ (to 0), which is accompanied by decrease (increase) of the radial edge length from $l_{{\text{c}}}$ to 0 ($2l_{{\text{c}}}$) so that the sum of the two remains constant and equal to $2l_{{\text{c}}}$. Hence the whole transformation of an isotropic structural element conserves the total length of the six hexagon sides and six radial edges equal $12l_{{\text{c}}}$.

The number of the isotropic structural elements, which can undergo such transformation independently of each other is $\frac{{N_{j}^{*} }}{8}$. Assuming that the number of the geometrical states corresponding to the whole range of the transformation of one isotropic structural element can be characterized by a discrete number, $m_{I}$, the total number of the system states of the same energy but different architecture, $M_{I}$, scales as

18 $$ M_{I} = \left( {m_{I} } \right)^{{\frac{{N_{j}^{*} }}{8}}} . $$

A simultaneous expansion of the hexagons of all $\left( {\frac{{N_{j}^{*} }}{8}} \right)$ independent isotropic structural elements to their maximum size ($2l_{{\text{c}}}$) results in a new hexagonal network with fewer but larger hexagonal cells whose sides consist of pair of bundles, which we will call the second order network. The isotropic transformation of the second order network generates an additional set of the minimal energy states whose number scales as

19 $$ \overline{{M_{I} }} \approx \left( {\overline{{m_{I} }} } \right)^{{\frac{{N_{j}^{*} }}{32}}} . $$

The number of states of the second order network is significantly smaller than that of the first one, $\overline{{M_{I} }} \ll M_{I}$. Analogously, further higher order networks get generated but add progressively little to the total number of the conformational states of the minimal energy, $F_{{\text{N}}}^{*}$. Thus, the number of the minimal energy states obtained through the isotropic transformation can be estimated with a good accuracy by (Eq. 18).

The telescopic transformation is based on a different type of the network structural elements referred below to as the linear structural element, which consists of a zigzag-like row of junctions connected by edges with additional side-edges emerging from every second junction perpendicularly to the zigzag axis, as illustrated in (Fig. 3C). The zigzag travers the whole network. There are three possible directions of the zigzag axis orientation. In the initial state the lengths of all the zigzag- and side-edges are equal to each other and to $l_{{\text{c}}}$. The telescopic transformation of a linear structural element consists in homogeneous extension or contraction of its side-edges (Fig. 3C). It can be easily seen that, provided that the network area is kept constant, the telescopic transformations of the linear structural elements do not change the overall bundle length. The number of the linear structural elements is proportional to the linear dimension of the network and, consequently, to $\sqrt {N_{j}^{*} }$. Assuming $m_{L}$ to be a discrete number of conformations, which can be adopted by one linear structural element through the telescopic transformation, the corresponding total number of the network conformations, $M_{L}$, is approximately given by:

20 $$ M_{L} \approx \left( {m_{L} } \right)^{{\sqrt {N_{j}^{*} } }} . $$

Comparing Eqs. (18) and (20), we conclude that for large networks, $N_{j}^{*} \gg 1$, the number of the minimal energy states corresponding to the transformations of the isotropic structural elements is much larger than that produced by the telescopic transformations. Thus, the most probable configurations of the network belong to those generated by the transformation of the isotropic structural elements (Fig. 3B).

It has to be noted that the stability conditions for the network configurations obtained through the described above transformations are different from that derived for homogenous hexagonal networks (Eq. 12). However, the principle remains the same, namely, any of the degenerated network configurations is stable if the junction folding rigidity, $f_{{\text{j}}}^{0}$, is sufficiently large compared to the absolute value of the tension, $\gamma$. In the case of isotropic structural element transformations, the condition guaranteeing stability of all configurations is $f_{{\text{j}}}^{0} > \frac{9}{10}\gamma b$, (see Supplementary Information).

### Irregular networks

Here we explore the possibility that irregular network configurations whose junctions, generally, deviate from the symmetric conformation, may have lower energies than those of the hexagonal networks with symmetric junctions analyzed above.

The network energy can be generally presented as:

21 $$ F_{{\text{N}}} \left( {N_{{\text{j}}} ,N_{{\text{M}}} ,\left\{ {\vec{r}^{\left( i \right)} } \right\},\left\{ {\phi_{1}^{\left( i \right)} ,\phi_{2}^{\left( i \right)} } \right\}} \right) = \mathop \sum \limits_{i = 1}^{N} f_{{\text{j}}}^{ } \left( {\phi_{1}^{\left( i \right)} ,\phi_{2}^{\left( i \right)} } \right) - \gamma L\left( {N_{{\text{j}}} ,N_{{\text{M}}} ,\left\{ {\vec{r}^{\left( i \right)} } \right\},\left\{ {\phi_{1}^{\left( i \right)} ,\phi_{2}^{\left( i \right)} } \right\}} \right), $$

where $N_{{\text{M}}}$ is the number of bundle source points on the boundary between the network and the reservoir, $\left\{ {\vec{r}^{\left( i \right)} } \right\}$ are the junction coordinates, and $\left\{ {\phi_{1}^{\left( i \right)} ,\phi_{2}^{\left( i \right)} } \right\}$ are the two independent angles defining the configuration of each three-way junction. The first and second contributions in the right-hand side of (Eq. 21) represent the total junction, $F_{{\text{j}}}$, and bundle, $F_{{\text{B}}}$, energies, respectively. The equation (Eq. 21) defines a complex potential energy surface in $4N_{{\text{j}}} + 2$ dimensional parameter space. Searching for the global energy minimum configuration of an irregular network with a given number of three-way junctions is an extremely difficult task even for a relatively small number of junctions.

To address this issue, we numerically generated irregular network configurations using random Voronoi tessellations^22^. To obtain each network configuration, a set of random seed points was generated, which defined the positions and the angles of $N_{{\text{j}}}$ three-way junctions within the network area, $A$, while the corresponding Voronoi tessellation generated the network edges. For each generated network, the energy was calculated according to (Eq. 21).

We used this numerical method to investigate irregular network configurations for different sets of the three independent system parameters: the number of junctions in the network $N_{{\text{j}}}$, the negative tension, -$\gamma$, and the characteristic length scale of the network, $l_{{\text{c}}}$, which is given by (Eq. 15) and equals the side length of the unit cell in the optimal hexagonal network.

For each set of the system parameters, we generated a large (~ 10^5^) but finite ensemble of irregular network configurations. The planar area, $A$, of each network realization, was taken to be $A = 15^{2}$ µm, matching the area of a central KIF of diameter $\sim 8.5$ µm. This selection of a specific area does not limit the generality of our results since all extensive values scale with the area, $A$.

**Figure 4:**
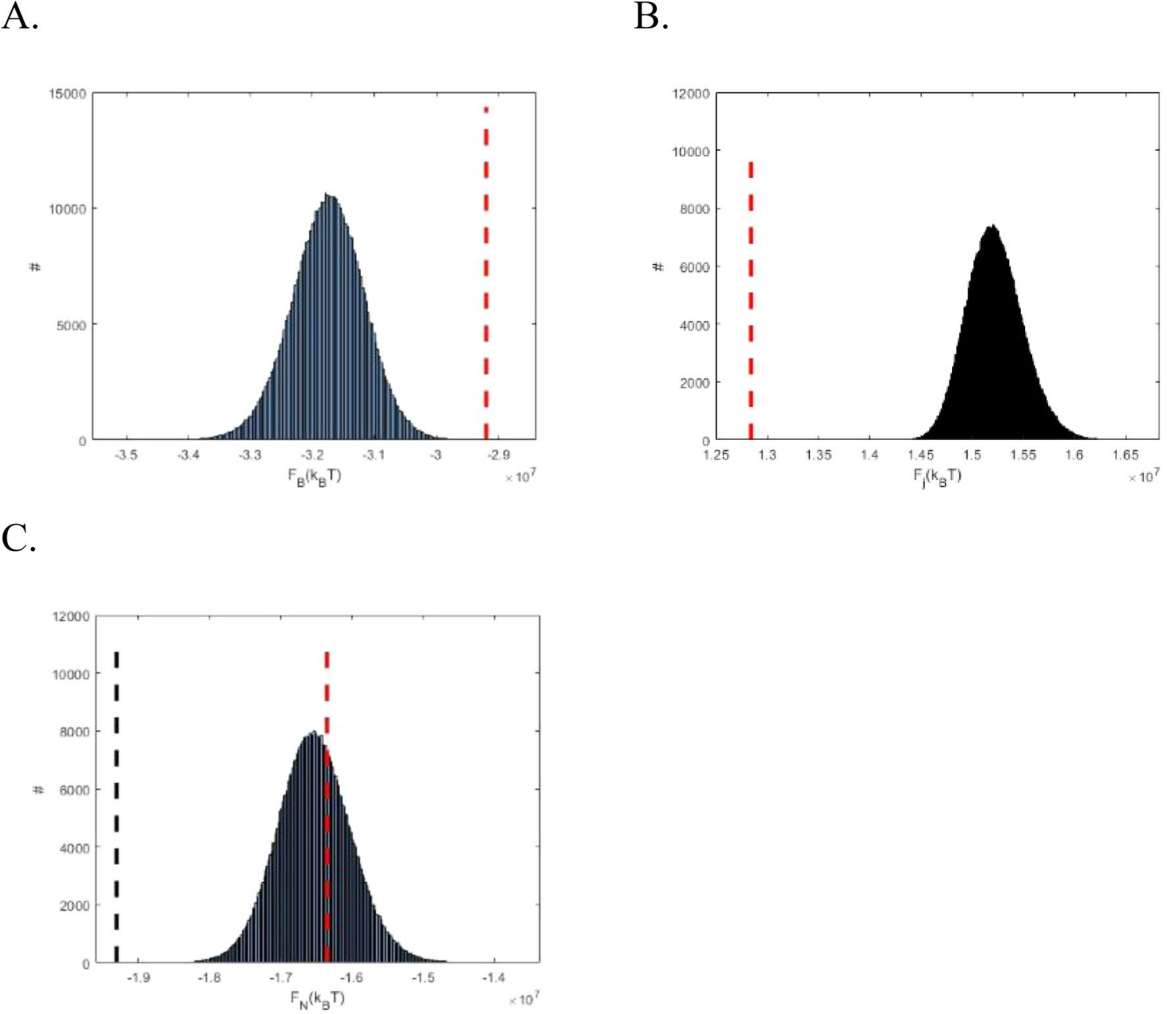
Histograms for distributions of the bundle energy (**A**) junction energy (**B**) and total network energy (**C**) calculated for the ensemble of the irregular network configurations generated by random Voronoi tessellations for the following values of parameters: $N_{{\text{j}}} = 77$, $N_{{\text{M}}} = 25$, $l_{{\text{c}}} = \frac{4}{3}$ µm and $\gamma = 170 \times 10^{3} {\text{k}}_{{\text{B}}} {\text{T }}$ µm^−1^. The vertical dashed red lines show the bundle, junction and total energies for the corresponding hexagonal network. The vertical dashed black line in panel (**C**) indicates the realization with the lowest total energy found in the ensemble.

An example of histograms representing the distributions of the bundle, $F_{{\text{B}}}$, junction, $F_{{\text{j}}}$, and total, $F_{{\text{N}}}$, energies among the ~ 10^5^ irregular configurations of the networks having $N_{{\text{j}}} = 77$ junctions and $N_{{\text{M}}} = 25$ bundle sources is given in (Fig. 4A–C). For comparison, the corresponding energies of the hexagonal network with symmetric junctions having the same numbers of $N_{{\text{j}}}$ and $N_{{\text{M}}}$ are shown by dashed vertical red lines.

The histograms are well described by Gaussian distribution functions, the widths of which are an order of magnitude smaller than the corresponding mean energies. The bundle energy, $F_{{\text{B}}}$, of the hexagonal network lies in the high-energy tail of the histogram for irregular networks (see (Fig. 4A), while the junction energy of the hexagonal network, $F_{{\text{j}}}$, obviously gives the low bound for $F_{{\text{j}}}$ of the irregular networks (see (Eq. 10) and (Fig. 4B). As a result, the total energy of the hexagonal network, $F_{{\text{N}}}$, is close to the mean value of the irregular network energy (Fig. 4C).

**Figure 5:**
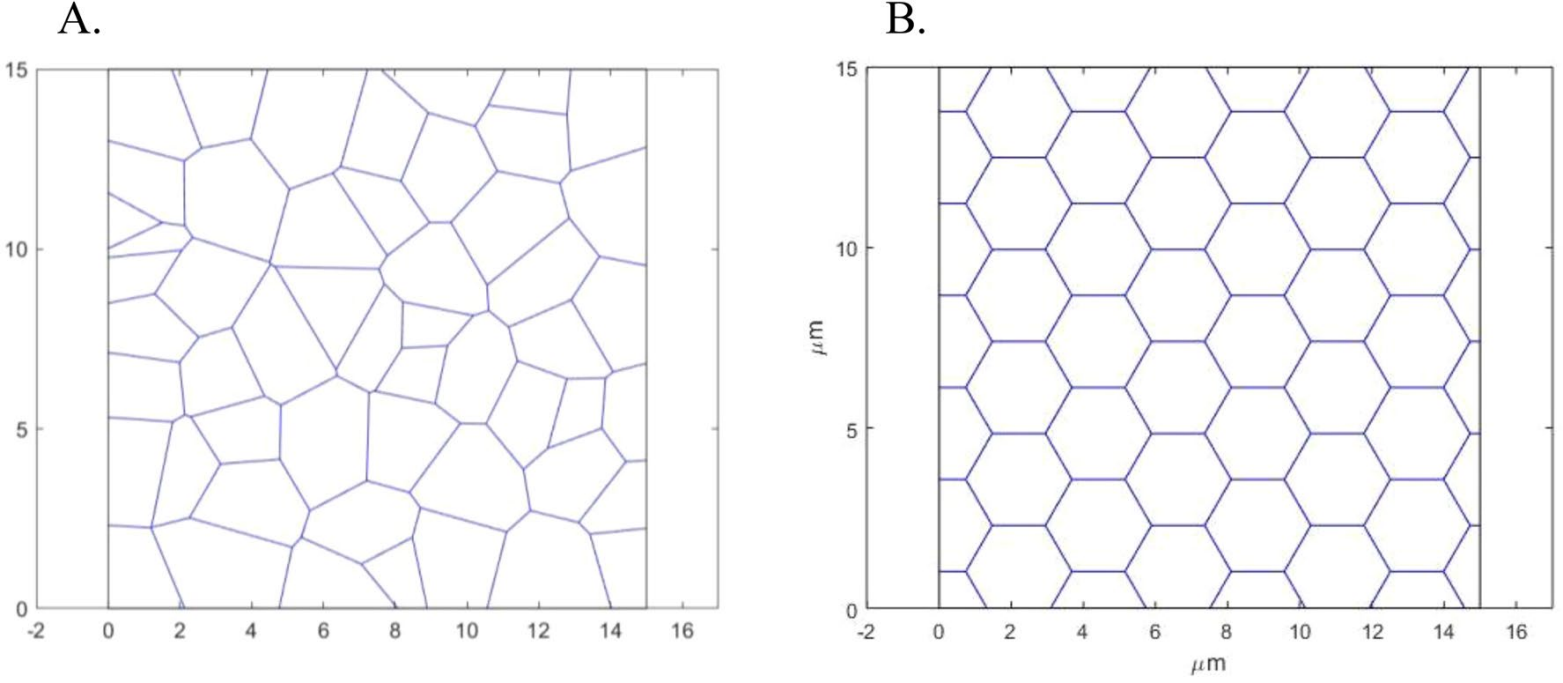
Optimal irregular (**A**) and the corresponding homogeneous hexagonal (**B**) network configurations for $N = 77$, $N_{{\text{M}}} = 25$, $l_{{\text{c}}} = 1$ µm, $\gamma = 170 \times 10^{3} {\text{k}}_{{\text{B}}} {\text{T}}$ µm^−1^.

As illustrated by (Fig. 4C), for the specific parameter set used in the computations, there are irregular network configurations whose total energy is lower than that of the hexagonal configuration with symmetric junctions. To generalize this conclusion, we picked the irregular configurations with the lowest total energy for all analyzed parameters sets. We refer to these configurations as the optimal configurations characterized by the energy $F_{{\text{N}}}^{0}$ for a given number of junctions. An example of an optimal configuration is presented in (Fig. 5A), whereas the hexagonal network with symmetric junctions having the same numbers of junctions, $N_{{\text{j}}}$, and bundle sources, $N_{{\text{M}}}$, is presented in (Fig. 5B), for comparison.

**Figure 6:**
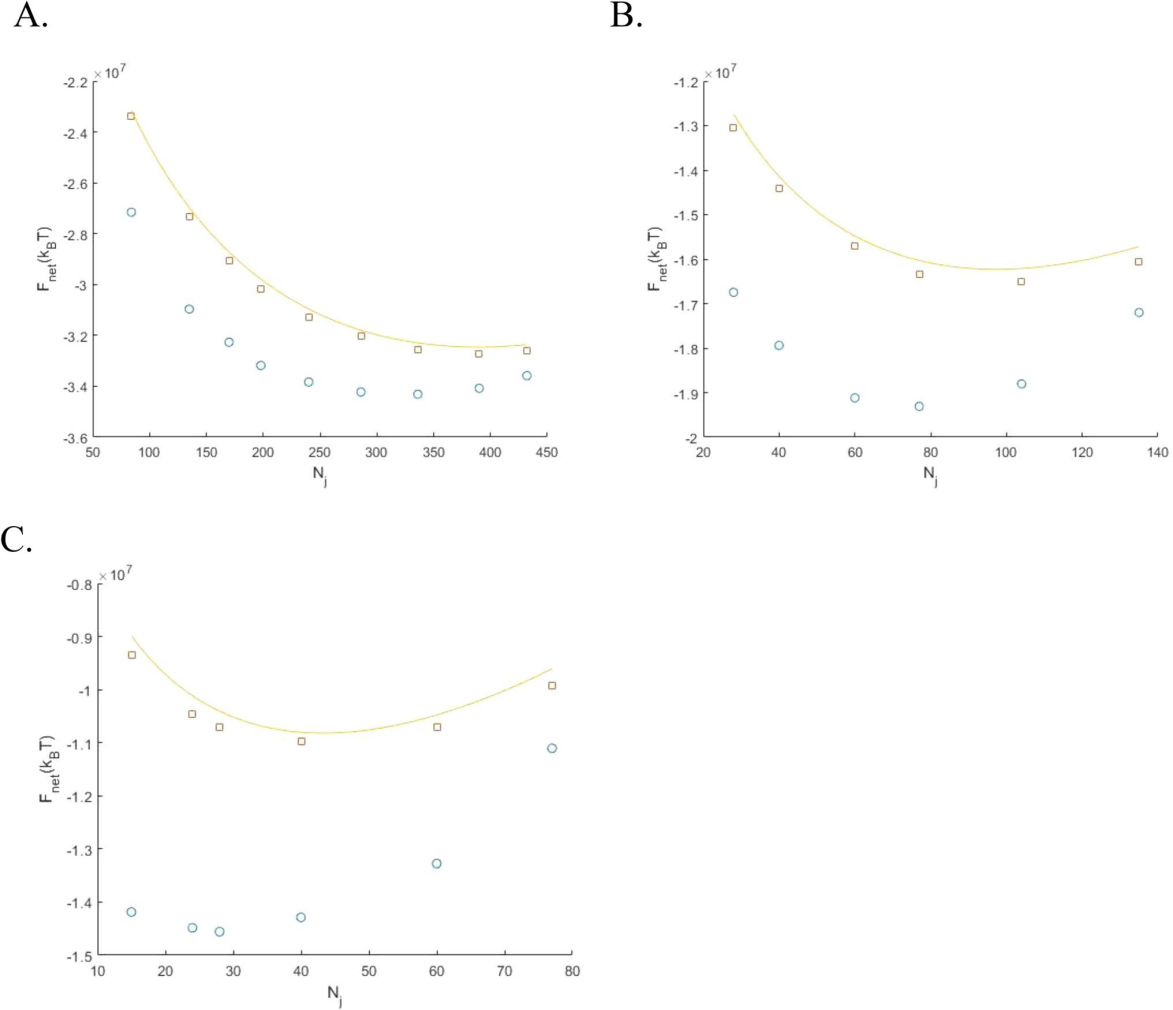
The total energy of the optimal irregular configuration (blue circles) and of the hexagonal configuration with symmetric junctions (red squares) as functions of the number of junctions. The number of sources, $N_{{\text{M}}}$, are equal for the hexagonal and irregular networks. The solid line shows the results of calculations according to the approximate analytical equation (Eq. 13) which neglects the contributions of the network edges connected to the boundary. The parameter values used are: $\gamma = 170 \times 10^{3} {\text{k}}_{{\text{B}}} {\text{T}}$ µm^−1^ and $l_{{\text{c}}} = \frac{2}{3}$ µm (**A**) $l_{{\text{c}}} = \frac{4}{3}$ µm (**B**) and, $l_{{\text{c}}} = 2$ µm (**C**).

**Figure 7:**
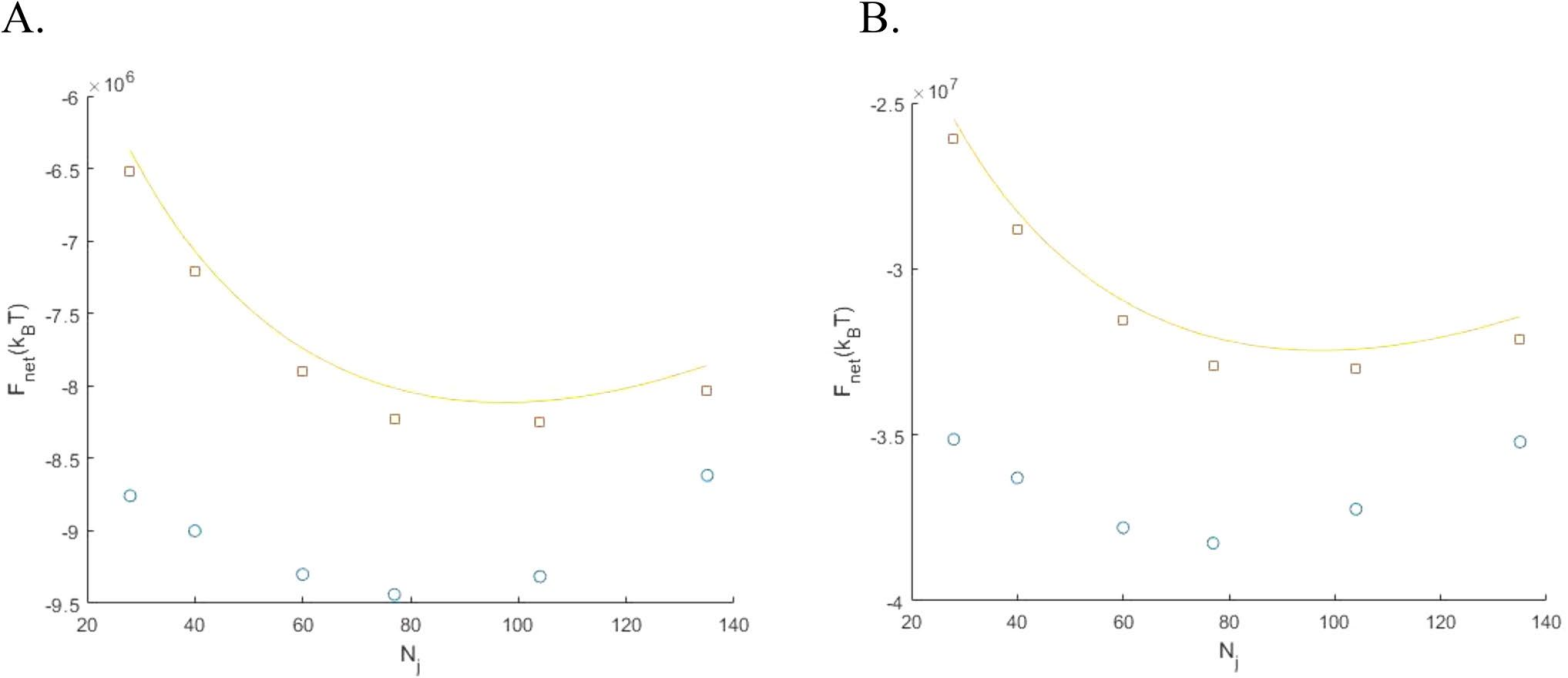
The total energy of the optimal irregular configuration (blue circles) and of the hexagonal configuration with symmetric junctions (red squares) as functions of the number of junctions. The number of sources, $N_{{\text{M}}}$, are equal for the hexagonal and irregular networks. The solid line shows the results of calculations according to the approximate analytical equation (Eq. 13) which neglects the contributions of the edges, connected to the boundary. The parameter values used are: $l_{{\text{c}}} = \frac{4}{3}\;$ µm and $\gamma = 85 \times 10^{3} {\text{k}}_{{\text{B}}} {\text{T}}$ µm^−1^ (**A**) and, $\gamma = 340 \times 10^{3} {\text{k}}_{{\text{B}}} {\text{T}}$ µm^−1^ (**B**).

The optimal configuration energy, $F_{{\text{N}}}^{0}$, as a function of number of junctions, $N_{{\text{j}}}$, for a set of different characteristic lengths, $l_{{\text{c}}}$, and different absolute values of tension, $\gamma$, are presented in (Figs. 6 and 7), respectively. For comparison, each figure presents by red squares the energies $F_{{\text{N}}}^{*}$ of the hexagonal configurations with the same number of junctions and source points. The solid line shows the results of calculations according to the approximate analytical equation (Eq. 13) which neglects the contributions of the network edges connected to the boundary. The latter is in an excellent agreement with the exact result for the hexagonal network (red squares).

For all studied sets of parameters, the energies of the optimal irregular configurations, $F_{{\text{N}}}^{0}$, are significantly lower than those of the hexagonal networks with symmetric junction, $F_{{\text{N}}}^{*}$. The reason for the predicted energetic favorability of the irregular network compared to the hexagonal one is a larger overall bundle length and the related energy contribution of the negative tension. In spite of the substantial difference in values, $F_{{\text{N}}}^{0}$ and $F_{{\text{N}}}^{*}$ exhibit similar dependencies on the number of junctions $N_{{\text{j}}}$, with minima corresponding to the optimal junction densities (Figs. 6 and 7). Notably, the number of junctions corresponding to the minimum of $F_{{\text{N}}}^{0}$, is independent of the tension, − $\gamma$, as predicted analytically for $F_{{\text{N}}}^{*}$ (Eq. 17).

Our simulations also showed that, as expected, also for the irregular network configurations the average dimension of the network unit-cell, $l^{*}$, corresponding to the minimum of energy as a function of number of junctions, is close the characteristic length, $l_{{\text{c}}}$, given by (Eq. 15). In particular, the results presented in (Figs. 6 and 7) show that $\frac{{l^{*} }}{{l_{{\text{c}}} }} \simeq$ 1.2 for $l_{{\text{c}}} = \frac{2}{3}, \frac{4}{3},$ and 2 µm. Thus, (Eq. 15) provides a good estimation for the characteristic unit-cell scale for both the irregular and the hexagonal network configurations.

**Figure 8:**
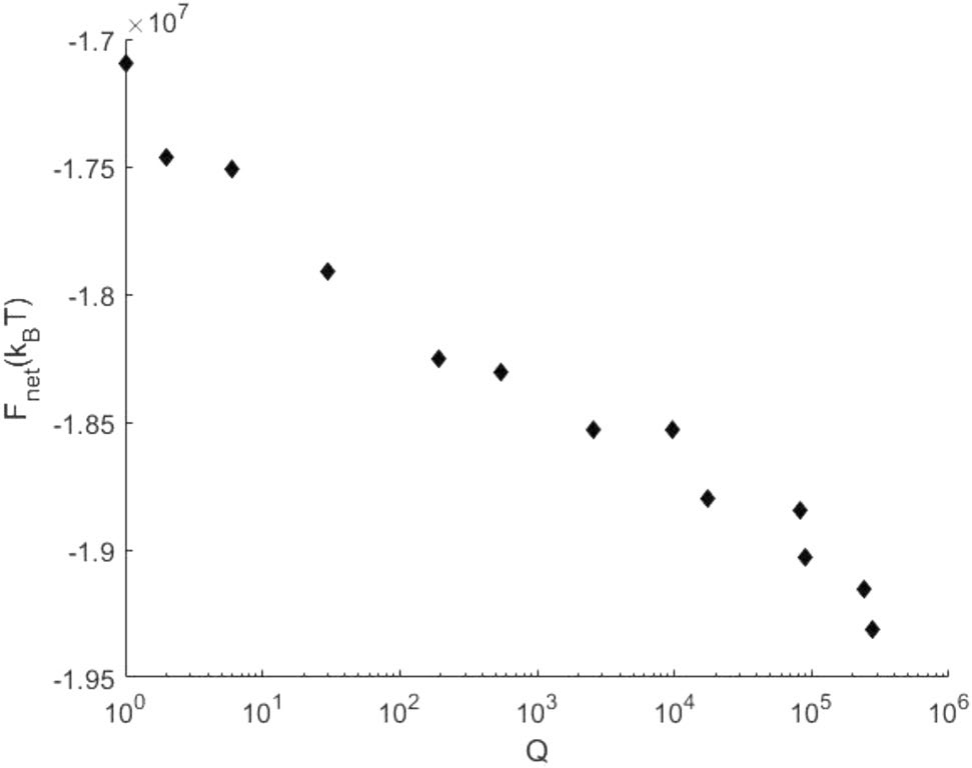
A semi-log plot illustrating the convergence of the lowest energy configuration using an ensemble of irregular realizations generated by random Voronoi tessellations. $Q$ is the number of generated realizations. The results were obtained for the same system parameter values as in (Fig. 4).

To assess the efficiency of these estimations, we evaluated the convergence of our numerical procedure with increase of the tessellation number $Q$. The results illustrated in (Fig. 8) show that the energy levels off exponentially with $Q$. Thus, we expect that the found minimal energies of the irregular configurations may serve as a good approximation of the global energy minima.

## Discussion

Here we analyzed the structures and stability of planar two-dimensional networks of bundles inter-connected by mobile three-way junctions. The special features of the considered networks, as compared to those investigated previously, is the negative value of the tension imposed on the system and the rigidity of the network junctions with respect to folding deformations. As implied by the existence of tension, the networks were assumed to be connected to an external reservoir of the bundle material. The energetically preferable network configurations were determined by an interplay between the system tendency to increase the overall length of the bundles driven by the negative tension and the energy cost of creation of the network junctions. We described these network configurations by the optimal values of the junction number, the overall bundle length, and by the network morphology.

As a paradigm of such system we used the networks of Keratin Intermediate Filaments observed in live cells^16^.

A general conclusion of our analysis is that the negative sign of the tension is mandatory for the existence of the system configurations stable with respect to the network withdrawal into the reservoir.

First, we examined analytically a limiting case of hexagonal networks with symmetric junctions for which all three inter-bundle angles within a junction equal $\frac{2\pi }{3}$. We determined the optimal network density and derived the conditions of the network stability with respect to folding of the junctions. We described the conformations degeneracy of the optimal network configurations.

Further, we studied, by numerical simulations based on the Voronoi tessellation method, the irregular network configurations, which had, generally, asymmetric three-way junctions. We demonstrated that such configurations exhibit significantly lower energies than the regular hexagonal ones. At the same time, the dependence of the minimal energy of the irregular configurations on the number of junctions was similar to that predicted analytically for the regular hexagonal configurations. Moreover, the unit-cell sizes of the optimal irregular configurations were close to those determined for the regular hexagonal configuration. Hence scale-wise the regular hexagonal network approximation provides a satisfactory description of the system.

### Limitations of the analysis

One limitation of our study is the use of the Voronoi tessellations algorithm for generation of the irregular network configurations. This method produces only a subset of configurations for which all inter-bundle angles in the junctions remain smaller than or equal to $\pi$. This restriction does not significantly affect the results if the energy cost of the junction deviations from the symmetric conformation is sufficiently large. To reliably fulfill this condition, we limited our computations by the range of system parameters satisfying Eq. (12), which can be rewritten in terms of $N_{{\text{j}}}$, $A$ and $l_{{\text{c}}}$ as

22 $$ N_{{\text{j}}} > \frac{{3^{5/2} }}{200} \frac{A}{{l_{{\text{c}}}^{2} }}. $$

The smallest values of $$N_{j}$$ satisfying the condition (Eq. 22), for the used value of the area, $$A = 15$$ µm^2^, are, approximately, $$8, 18$$, and $$70$$ for, respectively, $$l_{{\text{c}}} =$$ 1.5 µm, 1 µm and $$0.5 $$ µm. This condition determined the lower limits of the analyzed networks (Figs. 6 and 7).

Another minor limitation is related to our assumption that the KIF network plane is constrained to a flat surface. In reality, the network plane has a sphere-like shape, and undergoes small out-of-plane perturbations. According to our estimations, this does not change the qualitative conclusions of our study since the radius of the keratin network plane (~ 5 μm) is sufficiently large compared to the average length of bundles between consecutive junctions (~ 1 µm).

## Supplementary Information

Supplementary Information.
