# Supplementary material for: Negative tension controls stability and structure of intermediate filament networks
Source: Sci Rep. 2022 Jan 7;12:16. doi: 10.1038/s41598-021-02536-0 (PMC8741771; doi:10.1038/s41598-021-02536-0)
Supplement: Supplementary file 1 — Supplementary Information. [file 41598_2021_2536_MOESM1_ESM.docx]

**Supplementary information**

**Derivation of the stability condition for homogenous hexagonal networks**

Here we analyze the stability of a mechanical equilibrium state of a KIF network characterized by a homogenous hexagonal morphology. In order to determine the conditions for the stability of the network against an infinitesimal displacement of a junction, we will calculate the change in the network’s free energy to a leading order. To do that, we will use equations (Eqs. 1 and 10), presented in the main text, to calculate the decrease in bundling energy, associated with the negative tension and facilitated by an increase in the overall network length, compared to the increase in junctions’ energy, due to deviations from the preferred symmetric state, associated with the rigidity of junctions.

Starting from a homogenous hexagonal network, composed of identical hexagons of edge length $b$, we consider an infinitesimal displacement of a single junction, in a general direction. Let us use the notation depicted in (Supplementary Figure 1), denoting the magnitude of the infinitesimal displacement with $xb,$ where $x\ll1$ is a dimensionless parameter, and denoting the angle of the displacement vector direction, relative to a hexagon edge, with $\varphi$.


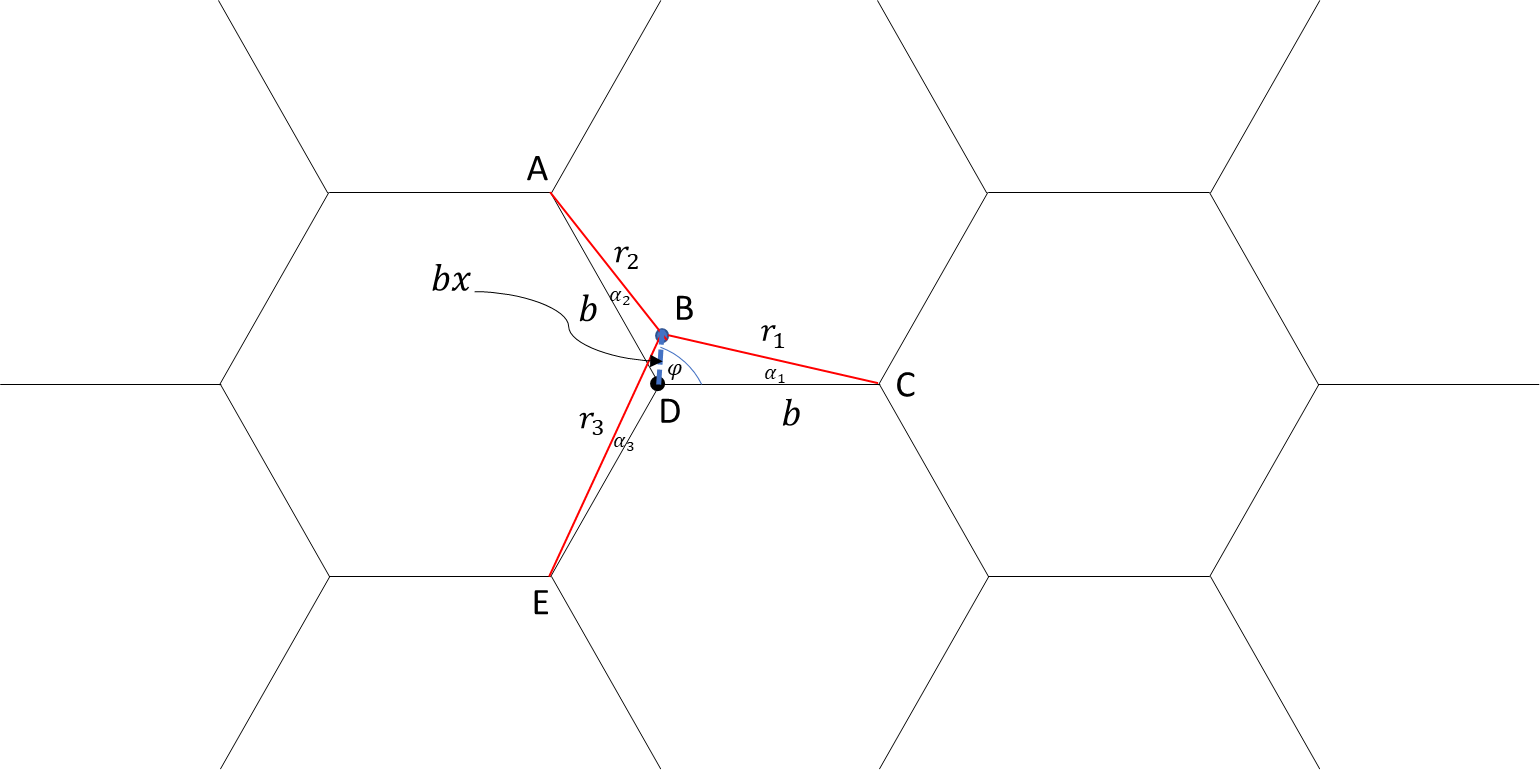


Supplementary Figure 1: A sketch illustrating an infinitesimal displacement of a junction from point D to point B, while keeping constant the positions of the junctions in C,E and A. The three red lines, which lengths are denoted with $\{{r\}}_{i}$, mark the changed network edges after the displacement of the junction. The angles formed between the changed edges and the original edges are denoted with $\left\{ \alpha\right\}_{i}$.

Considering the geometrical description and notations shown in (Supplementary Figure 1), the overall change in the network’s length, upon an infinitesimal displacement of a junction, is given by:

$\Delta L=r_{1}+r_{2}+r_{3}-3b$ (S1)

To determine the lengths, $\left\{ r \right\}_{i}$, of the changed edges, we use the cosine law in each of the triangles $\Delta DBC$, $\Delta ABD$ and $\Delta DBE$, which, consequently, gives the following three equations:

$r_{1}^{2}=b^{2}+\left( xb \right)^{2}-2xb^{2}\cos\varphi$ (S2)

$r_{2}^{2}=b^{2}+\left( xb \right)^{2}-2xb^{2}\cos\left( \frac{2\pi}{3}-\varphi\right)$ (S3)

$r_{3}^{2}=b^{2}+\left( xb \right)^{2}-2xb^{2}\cos\left( \frac{2\pi}{3}+\varphi\right)$ (S4)

Plugging (Eqs. S2-S4) into (Eq. S1), and expanding it in a power series for $x\ll1$ to a leading order gives:

$\Delta L\simeq\frac{3}{4}x^{2}b$ (S5)

Interestingly, the length always increases, irrespective of the direction of displacement. The angle $\varphi$ enters only at higher orders in the expansion which are not relevant for the stability analysis.

Consequently, using (Eq. 1) from the main text, we get a bundling energy gain of:

$-\gamma\Delta L\simeq-\frac{3}{4}x^{2}\gamma b$ (S6)

where $-\gamma$ is the negative tension in network, as introduced in the main text.

Next, we calculate the elastic (bending) energy associated with the change of junctions’ conformation relative to their preferred symmetric state. As shown by (Eq. 7) in the main text, the energy of an optimal-size junction is a function of two independent angles $\phi_{1}$ and $\phi_{2}$ opened between a pair of consecutive bundles connected by the junction. Referring to the geometry shown in (Fig. S1), upon the infinitesimal displacement, there are four junctions which change their symmetric configuration: the junction that was displaced and the three junctions connected to it by the changed network edges.

By finding the angles of each changed junction, expressing them with the dimensionless parameter, $x$, and using the series expansion (Eq. 10) presented in the main text, we evaluated the change in energy for each junction. The total change in junctions’ energy, expanded to a leading order, is given by:

$\frac{5}{3}f_{j}^{0}x^{2} ,$ (S7)

which is, again, independent of the displacement angle $\varphi$ to a leading order.

The sum of (Eq. S6 and S7) gives the total change in the free energy of the network, expanded to a leading order:

$\Delta F\simeq\left( \frac{5}{3}f_{j}^{0}-\frac{3}{4}\gamma b \right)x^{2} .$ (S8)

Inspection of (Eq. S8) shows that a positive change in free energy, which commensurate to a stable equilibrium, is obtained for the condition:

$f_{j}^{0}>\frac{9}{20}\gamma b$ (S9)

**Derivation of the stability condition for non-homogenous hexagonal networks acquired by transformations of isotropic structural elements**

Here we analyze the stability of a mechanical equilibrium state of a KIF network characterized by a non-homogenous hexagonal morphology. Specifically, let us start from a homogenous network of hexagons, of side length $b$, and then expand/contract just one isotropic structural element (see Supplementary Figure 2). The different lengths of the expanded structural element are a function of a dimensionless parameter, $\beta$, which can vary between 0 (full expansion) and 2 (full contraction), as seen in (Supplementary Figure 2). Similar to the process considered in Supplementary Note 1, in order to determine the conditions for the stability of the network against an infinitesimal displacement of a junction, we will calculate the change in the network’s free energy to a leading order by using (Eqs. 1 and 10).

Let us consider an infinitesimal displacement of a single junction, in a general direction as shown in (Supplementary Figure 2). Following the notation depicted in (Supplementary Figure 2), we denote the magnitude of the infinitesimal displacement with $xb,$ where $x\ll1$ is a dimensionless parameter, and denoting the angle of the displacement vector direction, relative to a hexagon edge, with $\varphi$.


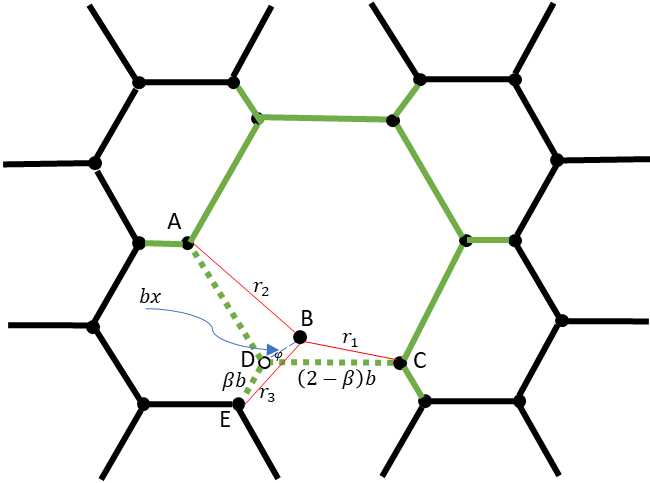


Supplementary Figure 2: A sketch illustrating an infinitesimal displacement of a single junction from a state of one expanded isotropic structural element. The lengths of the structural element are governed by the parameter $\beta$, which can freely vary from 0 to 2. The displacement of the single junction was performed from point D to point B, while keeping constant the positions of the junctions in C, E and A. The three red lines, which lengths are denoted with $\{{r\}}_{i}$, mark the changed network edges after the displacement of the junction.

The overall change in the network’s length, upon an infinitesimal displacement of a junction, is given by:

$\Delta L=r_{1}+r_{2}+r_{3}-\left( 4-\beta\right)b$ (S10)

To determine the lengths, $\left\{ r \right\}_{i}$, of the changed edges, we use the cosine law in each of the triangles $\Delta DBC$, $\Delta ABD$ and $\Delta DBE$, which, consequently, gives the following three equations:

$r_{1}^{2}=\left( \left( 2-\beta\right)b \right)^{2}+\left( xb \right)^{2}-2x\left( 2-\beta\right)b^{2}\cos\varphi$ (S11)

$r_{2}^{2}=\left( \left( 2-\beta\right)b \right)^{2}+\left( xb \right)^{2}-2x\left( 2-\beta\right)b^{2}\cos\left( \frac{2\pi}{3}-\varphi\right)$ (S12)

$r_{3}^{2}={(\beta b)}^{2}+\left( xb \right)^{2}-2x\beta b^{2}\cos\left( \frac{2\pi}{3}+\varphi\right)$ (S13)

Plugging (Eqs. S11-S13) into (Eq. S10), and expanding it in a power series for $x\ll1$ to a leading order gives:

$\frac{\Delta L}{b}\simeq\frac{2+\beta+2\left( \beta-1 \right)\cos\left( 2\left( \frac{\pi}{3}-\varphi\right) \right)}{4\beta\left( 2-\beta\right)}x^{2}$ (S14)

Notice that (Eq. S14) depends on the angle of displacement $\varphi$, in contrast to (Eq. S5). This is due to the inflation of one hexagon which breaks the symmetry present in the homogenous hexagonal network case. It should also be noted that for $\beta=1$, (Eq. S14) collapses into (Eq. S5), as expected. Regardless of the value picked for $\varphi$, we note that the change in length is always positive.

Next, we calculate the bending energy associated with the change of junctions’ conformation relative to their preferred symmetric state. By finding the angles of each changed junction, expressing them with the dimensionless parameter, $x$, and using the series expansion (Eq. 10) presented in the main text, we evaluated the change in energy for each junction. The total change in junctions’ energy, expanded to a leading order, is given by:

$f_{j}^{0}\frac{16+\beta(11\beta-12)+4\left( \beta-1 \right)\left( \beta+4 \right)\cos\left( 2\left( \frac{\pi}{3}-\varphi\right) \right)}{9{(2-\beta)}^{2}\beta^{2}}x^{2} ,$ (S15)

It should be noted that (Eq. 15) depends on the angle of displacement, $\varphi$, in contrast to (Eq. S7), which was derived for the case of homogenous hexagonal network. This is due to the break of symmetry discussed above. Again, for $\beta=1$, (Eq. S15) collapses into (Eq. S7), as expected. Moreover, regardless of the exact values picked for $\beta$ and $\varphi$, the change in junctions’ energy shown in (Eq. S15) is always positive, as required.

The sum of (Eq. S14 and S15) gives the total change in the free energy of the network, expanded to a leading order:

$\Delta F\simeq\left( \frac{16+\beta(11\beta-12)+4\left( \beta-1 \right)\left( \beta+4 \right)\cos\left( 2\left( \frac{\pi}{3}-\varphi\right) \right)}{9{(2-\beta)}^{2}\beta^{2}}f_{j}^{0}-\frac{2+\beta+2\left( \beta-1 \right)\cos\left( 2\left( \frac{\pi}{3}-\varphi\right) \right)}{4\beta\left( 2-\beta\right)}\gamma b \right)x^{2} .$ (S16)

Inspection of (Eq. S16) shows that a positive change in free energy, which commensurate to a stable equilibrium, is obtained for the condition:

$\frac{f_{j}^{0}}{\gamma b}>\frac{9}{4}\beta\left( 2-\beta\right)\frac{2+\beta+2\left( \beta-1 \right)\cos\left( 2\left( \frac{\pi}{3}-\varphi\right) \right)}{16+\beta(11\beta-12)+4\left( \beta-1 \right)\left( \beta+4 \right)\cos\left( 2\left( \frac{\pi}{3}-\varphi\right) \right)}$ (S17)

The right-hand side of (Eq. S17) is a two-dimensional function of $\beta$ and $\varphi$, which has a global maximum value of $\frac{9}{10}$ for $\beta=0$ and $\varphi=\frac{\pi}{3}$. This global maximum value serves as an upper limit for the condition in (Eq. S17).
